# Assessment of CO_2_ biofixation and bioenergy potential of microalga *Gonium pectorale* through its biomass pyrolysis, and elucidation of pyrolysis reaction *via* kinetics modeling and artificial neural network

**DOI:** 10.3389/fbioe.2022.925391

**Published:** 2022-08-18

**Authors:** Ahmed Altriki, Imtiaz Ali, Shaikh Abdur Razzak, Irshad Ahmad, Wasif Farooq

**Affiliations:** ^1^ Department of Chemical Engineering, King Fahd University of Petroleum and Minerals, Dhahran, Saudi Arabia; ^2^ Department of Chemical and Materials Engineering, King Abdulaziz University, Rabigh, Saudi Arabia; ^3^ Interdisciplinary Research Center for Membranes and Water Security, King Fahd University of Petroleum and Minerals (KFUPM), Dhahran, Saudi Arabia; ^4^ Department of Bioengineering, King Fahd University of Petroleum and Minerals, Dhahran, Saudi Arabia

**Keywords:** microalgae, CO_2_ bio-fixation, biomass pyrolysis, thermogravimetric analysis, reaction kinetics

## Abstract

This study investigates CO_2_ biofixation and pyrolytic kinetics of microalga *G. pectorale* using model-fitting and model-free methods. Microalga was grown in two different media. The highest rate of CO_2_ fixation (0.130 g/L/day) was observed at a CO_2_ concentration of 2%. The pyrokinetics of the biomass was performed by a thermogravimetric analyzer (TGA). Thermogravimetric (TG) and derivative thermogravimetric (DTG) curves at 5, 10 and 20°C/min indicated the presence of multiple peaks in the active pyrolysis zones. The activation energy was calculated by different model-free methods such as Friedman, Flynn-Wall-Ozawa (FWO), Kissinger-Akahira-Sunose (KAS), and Popescu. The obtained activation energy which are 61.7–287 kJ/mol using Friedman, 40.6–262 kJ/mol using FWO, 35–262 kJ/mol using KAS, and 66.4–255 kJ/mol using Popescu showed good agreement with the experimental values with higher than 0.96 determination coefficient (R^2^). Moreover, it was found that the most probable reaction mechanism for *G. pectorale* pyrolysis was a third-order function. Furthermore, the multilayer perceptron-based artificial neural network (MLP-ANN) regression model of the 4-10-1 architecture demonstrated excellent agreement with the experimental values of the thermal decomposition of the *G. pectoral.* Therefore, the study suggests that the MLP-ANN regression model could be utilized to predict thermogravimetric parameters.

## 1 Introduction

Microalgae are potential candidates for CO_2_ biofixation and renewable energy. Microalgae can be cultivated in a closed photobioreactor located on non-arable land or in an open vast pond. It is a cost-effective source of carbon dioxide (CO_2_) mitigation through photosynthesis (Aresta et al., 2005). The fixation of CO_2_ by microalgae is sought as an attractive strategy to produce biofuels, aquaculture products, and renewable food. Furthermore, it can be processed into a variety of products, for example, fuel gases, soil modifiers, biodiesel, green diesel, and methane (Christenson and Sims, 2011). The fixation of CO_2_ by microalgae depends on various biotic and abiotic factors, such as temperature, quality and quantity of light, pH of the solution, mode of cultivation, purity, and CO_2_ concentration being supplied. Furthermore, CO_2_ fixation is also affected by microalgae strain (Daneshvar et al., 2022; Farooq et al., 2022). Microalgae biomass after CO_2_ fixation can be processed as an environmentally friendly renewable feedstock through different conversion technologies such as gasification, pyrolysis, liquefaction and bioethanol technology. Pyrolysis technology is a distinctive chemical reaction that produces valuable chemicals such as biochar, light olefins, and syngas. The pyrolysis of microalgae comprises numerous reactions in series and parallel. There are different pyrolysis processes for microalgae such as fast pyrolysis, slow pyrolysis, catalytic pyrolysis, and microwave-assisted pyrolysis (Farooq et al., 2021).

TGA examines the decomposition of materials by weight changes as a function of rising temperature. The relationship between temperature and weight loss due to oxidation, dehydration, and decomposition is recorded as the TGA plot, while derivative thermogravimetric (DTG) curve records the derivative of weight change with respect to temperature. The rate of the thermochemical reaction is represented by the peak of the DTG curve. The elevation of the DTG curve detects the potential to release volatile substances from a reaction during the slow process of pyrolysis (Yang et al., 2014). However, TGA technology only evaluates total weight loss due to reactions, restricts its usage, and provides general information on the overall kinetics of the reaction rather than individual reactions (Yang et al., 2019).

So far, commercialization of microalgae pyrolysis has been tested only on a bench scale although microalgae pyrolysis has been used since the early 1990s. TGA was extensively used to show the kinetics of the degradation process and to simulate the slow pyrolysis process (heating rate is usually lower than 1°C/s in a fixed-bed tubular reactor). Much research on pyrolysis of microalgae was conducted using a slow pyrolysis approach, while only a few studies were conducted using fast pyrolysis. Fast pyrolysis with a heating rate greater than 10°C/s can be performed in the fluidized bed reactor (Yang et al., 2019).

The kinetic data of TGA can be analyzed by various methods. The two most commonly applied approaches are model-free and model-fitting. The model-fitting method cannot characterize non-isothermal data adequately compared to the model-free approach. The model-free approach is much simpler, it avoids errors related to the kinetic model choice and offers better fitting of thermogravimetric curves than the model-fitting method. One of the limitations of this method is that several kinetic curves are collected to perform the analysis. Reaction rates can be calculated based on data collected at different heating rates at the same conversion value, resulting in the calculation of the activation energy at each conversion point (Subagyono et al., 2021). No study on thermogravimetric analysis of *G. pectorale is reported.* Fewer studies on the genetic transformation of *G. pectorale microalgae are available* (Lerche and Hallmann, 2009; Hanschen et al., 2016)*.* Lot of work has been done on CO_2_ fixation of microalgae along with its bioenergy potential. Besides improving the cultivation and harvesting process to increase the yield of biomass, a search for new strain with better growth and CO_2_ fixation potential is needed as well. To best of our knowledge, this strain has not been investigated for its CO_2_ fixation potential and its bioenergy contents. We targeted this strain as there is no study on potential of the strain for CO_2_ and bioenergy potential.

The objectives of this study were to 1) Investigate the potential of the *G. pectorale* microalga for its CO_2_ fixation potential, which to best of our knowledge has not been reported in the literature, 2) Estimate its total energy content through higher heating value (HHV), and 3) Kinetic analysis of the conversion process using model-free and model-fitting methods, and 4) The conversion of *G. pectorale* was modeled using an artificial neural network (ANN) to predict the pyrolysis behavior. The input data were time, temperature, and weight loss, while the output data was the change in weight loss with respect to time.

## 2 Materials and methods

### 2.1 Media preparations

The microalga was cultivated in two different growth mediums, namely Tris-Acetate-Phosphate (TAP) medium and Modified Bold 3N medium (3NBBM). To test whether this microalga prefers organic carbon or not, the first medium contains an organic carbon compound (CH_3_COOH) while the latter does not. Stock solutions for the growth medium were prepared in a 250 ml volumetric flask. After that, the stock solutions were combined in a 2 L flask for the growth medium. Each prepared medium was autoclaved at 121°C for 4 h to eliminate bacterial contamination and then allowed to cool to room temperature before microalgae inoculation.

### 2.2 *G. pectorale* biomass growth and CO_2_ fixation potential

*G. pectorale* (strain K3-F3–4, mating type minus, NIES-2863 obtained from the Microbial Culture Collection at the National Institute for Environmental Studies, Tsukuba, Japan; Available online: http://mcc.nies.go.jp/) was grown under continuous light (1,300 lux) in 50 ml of modified Bold’s 3N medium (UTEX, Austin, TX, United States) (Guerriero et al., 2018). The Erlenmeyer flasks were incubated at 25°C and 120 RPM on a shaker under approximate illumination of 100 μmol m^−2^ s^−1^ using cool white florescent light (Liu et al., 2019). Then, both the optical density (OD) and dry weights were taken daily. After 14 days of incubation with a continuous light supply, algal biomass was harvested, dried, and then ground to powder. A standard calibration curve for *G. pectorale was generated* to estimate its biomass concentration in [g/L] at any measured OD by Eq. 1.

$$DW\left[g/L\right]=0.263OD_{688}+0.0104$$ (1)

CO_2_ bio-fixation rate can be measured using the equation of the reference (Adamczyk et al., 2016).

$$R_{CO_{2}}=P\cdot C_{C}\frac{MW_{CO_{2}}}{MW_{C}}$$ (2)

where $R_{CO_{2}}$ is the fixation rate, and P is the productivity in [mg per L per Day], $C_{C}$ is the average carbon content calculated by the elemental analyzer, $MW_{C}$ is the molecular weight of one carbon atom, and $MW_{CO_{2}}$ is the molecular weight of CO_2_.

### 2.3 Ultimate analysis of *G. pectorale* and higher heating value

The ultimate analysis of the dried biomass was performed using (Perkin Elmer Model 2400 CHNS/O Elemental analyzer, Perkin Elmer Corporation). The harvested samples were dried in the drying oven at 60°C for 24 h. The dried biomass samples were weighted (0.75–1.5 mg) in clean tin capsules (5 mm × 8 mm, Perkin Elmer). The capsules were then heated to 975°C using oxygen gas as the combustion gas feed and helium gas as the purging gas in a furnace. The instrument was calibrated with different criteria of ±3.75 for hydrogen, ±0.15 for carbon and ±0.16 for nitrogen. Furthermore, the oxygen content was found by difference.

The higher heating value (HHV) was calculated from the equation of reference (Noushabadi et al., 2021).

$$\begin{array}{c} HHV\;\left[MJ/kg\right]=-0.8738\times N\times H^{-1.3101}\\ -0.1583\times C\times O^{0.3497}\\ +0.3856\times C\times {\left(H\times O\right)}^{0.1462}\\ +2.1436\times {\left(\frac{H}{O}\right)}^{-0.3846}\\ +0.1076\times C\times H^{-0.3846}+0.1098\times N\times S\\ -11.2794\times \left(\frac{H}{C}\right) \end{array}$$ (3)

where *C*, *H*, *N*, *O,* and *S* represent carbon, hydrogen, nitrogen, oxygen, and sulfur contents, respectively.

### 2.4 Thermogravimetric analysis of *G. pectorale* biomass

TGA analysis was conducted using the SDT Q600 TG-DTA thermogravimetric analyzer to explore the non-isothermal pyrolysis of microalgae remnants. Approximately 5–6 mg of each dried sample were placed in an alumina crucible, which was then inserted into the analyzer chamber. Nitrogen was continuously supplied with a constant flow of 100 ml/min as a purging gas to prevent undesirable oxidation reactions and remove any trapping gases. At first, the temperature for each run was equilibrated at 30°C. The sample was then heated from 30 to 800°C, with a heating rate of 5°C per minute. Additionally, two more heating rates at 10 and 20°C per minute were employed using the same procedure.

### 2.5 Kinetic analysis

The kinetics of chemical reactions can easily be determined from DSC or TGA measurements (Vyazovkin, 2020). The basic rate equation for this kinetic analysis is given by Eq. 4.

$$\frac{d\alpha }{dt}=k\left(T\right)\cdot f\left(\alpha \right)$$ (4)

where k(T) is the rate constant, f(α) is the kinetic model, and α is the degree of conversion, which is calculated by Eq. 5

$$\alpha =\frac{w_{0}-w}{w_{0}-w_{f}}$$ (5)

In which $w_{0},\;w$ , and $w_{f}$ are the initial, instantaneous, and the remaining mass of the sample, respectively.

With Arrhenius equation

$$k\left(T\right)=A\exp\left(\frac{-E_{a}}{RT}\right)$$ (6)

where A, *E*_*a*_, R, and T are the pre-exponential factor, activation energy, gas constant, and temperature.

The term k(T) in Eq. 4 is combinable with Eq. 6 and gives the equation below

$$\frac{d\alpha }{dt}=A\exp\left(\frac{-E_{a}}{RT}\right)\cdot f\left(\alpha \right)$$ (7)

Incorporating the temperature dependence of the reaction from the Arrhenius law and subsequently modifying it for isochronal heating, the equation becomes the following.

$$\frac{d\alpha }{dT}=\frac{A}{\beta }\cdot \exp\left(\frac{-E_{a}}{RT}\right)\cdot f\left(\alpha \right)$$ (8)

In which β is the applied heating rate $\left(\frac{dT}{dt}\right)$

After integration, Eq. 8 becomes:

$$g\left(\alpha \right)=\int_{0}^{\alpha }\frac{d\alpha }{f\left(\alpha \right)}=\int_{T_{0}}^{T}\frac{A}{\beta }\cdot \exp\left(\frac{-E_{a}}{RT}\right)\cdot dT$$ (9)

where g(α) is the integral form of f(α) and $T_{0}$ is the initial temperature.

A model-free technique will be adopted to estimate the activation energy.

#### 2.5.1 Model-free kinetics

Model-free kinetics assumes that the activation energy changes during the reaction. Furthermore, this approach also assumes that the activation energy at a particular conversion point is independent of temperature (“isoconversion principle”). Various model-free kinetic approaches are reported. The model-free approach allows one to determine the activation energy of a reaction without assuming a kinetic model. Various model-free kinetics approaches are reported. Friedman is a differential isoconversional method, whereas Ozawa-Flynn-Wall (OFW) and Kissinger-Akahira-Sunose (KAS) are integral isoconversional methods. In all methods, the measurements are analyzed for multiple conversion levels. These methods are suitable for multistep reactions and give an average activation energy value (Naqvi et al., 2018). Friedman requires at least two measurements.

Friedman (Friedman, 2007):

$$\ln\left(\beta \frac{d\alpha }{dT}\right)=\ln\left(\frac{d\alpha }{dt}\right)=\ln\left[A\cdot f\left(\alpha \right)\right]-\frac{E_{a}}{RT}$$ (10)

E_a_ is determined from the slope of $\ln\left(\frac{d\alpha }{dt}\right)\mathrm{versus}\frac{1}{T}$ plot at constant *α*.

OFW (Maia and de Morais, 2016):

$$\ln\beta =\mathrm{constant}-1.052\frac{E_{a}}{RT_{\alpha }}$$ (11)

KAS (Naqvi et al., 2018):

$$\ln\frac{\beta }{T_{\alpha }^{2}}\;=\mathrm{constant}-\frac{E_{a}}{RT_{\alpha }}$$ (12)

Popescu (Kokalj et al., 2017):

$$\ln\left(\frac{\beta }{T_{\alpha }-T_{\alpha -\Delta \alpha }}\right)=\mathrm{constant}-\frac{2E_{a}}{R\left(T_{\alpha }+T_{\alpha -\Delta \alpha }\right)}$$ (13)

where *∆α* is the conversion interval, *T*_*(α-∆α)*_ is the absolute temperature at *α-∆α*, and *T*_*α*_ is the temperature corresponding to *α*.

#### 2.5.2 Model-fitting kinetics

The model function in the rate equation can be attained by the linear regression of the equation below, which is known as the combined kinetics method (Pérez-Maqueda et al., 2006).

$$\ln\left(\frac{d\alpha }{dt}\right)-\text{ln}\left[{\left(1-\alpha \right)}^{n}\alpha^{m}\right]=\ln\left(cA\right)-\frac{E_{a}}{RT_{\alpha }}$$ (14)

where c, n, and m are the parameters of the model function, $f\left(\alpha \right)=c{\left(1-\alpha \right)}^{n}\alpha^{m}$

#### 2.5.3 Thermodynamic analysis

Thermodynamic parameters such as changes in the Gibbs free energy of activation, the enthalpy of activation, and the entropy of activation are obtained from the kinetic parameters by the equations below.

$$\Delta H=E_{a}-RT$$ (15)

$$\Delta G=E_{a}+RT_{p}\ln\left(\frac{K_{B}T_{p}}{hA}\right)$$ (16)

$$\Delta S=\frac{\Delta H-\Delta G}{T_{p}}$$ (17)

where K_B_ is the Boltzmann constant (1.381 × 10^−23^ J/K), h is the Planck constant (6.626 × 10^−34^ J s), and T_p_ is the temperature corresponding to the maximum DTG in Kelvin.

### 2.6 Artificial neural network

In this study, artificial neural network (ANN) models were developed to predict activation energy. ANN use the input/target data to map the patterns between the variables. Statistica 13.5 was used to develop the network architecture using MLP regression to model the target values. Temperature (K), heating rate (°C/min), conversion (−) and conversion rate (s^−1^) were used as input neurons in the input layer, while the activation energy was used as output neuron in the output layer. Three subsets were obtained randomly from the original data set as training (70%), testing (15%) and validation (15%). The Broyden–Fletcher–Goldfarb–Shanno (BFGS) algorithms were used to develop MLP based ANN regression models. The hidden and output layers use different built-in activation functions such as logistic, exponential, tangent hyperbolic, SoftMax, sine and gaussian. Each neuron is connected through its nodes to all nodes in the other layers with some network parameters (weights and biases). Neural networks are trained through supervised learning by minimizing the sum of squared errors. Trained networks were evaluated and verified using testing and validation data sets. Five best-performing models are retained, out of which one best is chosen using external validation and predictions.

## 3 Results and discussion

### 3.1 *G. pectorale* growth

**FIGURE 1:**
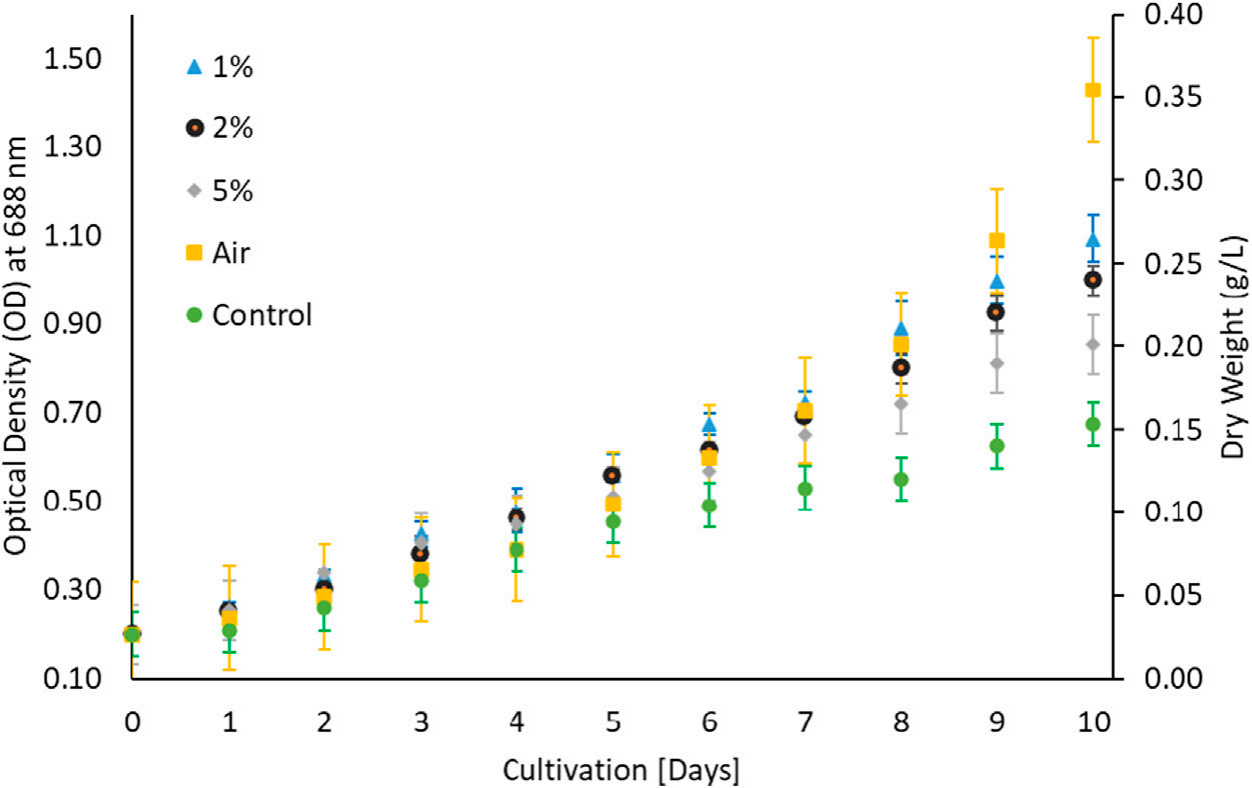
Different CO_2_ concentrations in 3NBBM.

Microalga *G. pectorale* was cultivated under standard room conditions in a modified Bold 3N medium with initial pH of 6.8 at different CO_2_ concentrations; 1, 2 and 5%. A maximum of 5% CO_2_ concentration was investigated, as some studies suggest that exceeding this concentration can harm algal cells and hinder their growth (Farooq, 2022; Farooq et al., 2022). Furthermore, *N. oculate* and *Chlorella* sp. strains have shown optimal growth at a concentration of 2% CO_2_ (Farooq et al., 2022). A light intensity of 100 μmol m^−2^ s^−1^ using cool white florescent light was used for the growth analysis. The effect of CO_2_ concentration on *G. pectorale* microalgal growth was found to be inversely proportional (Figure 1). Preference of microalgae for carbon source either inorganic as CO_2_ and organic carbon depends on algal species (Khan et al., 2022). There is no published study on this strain, that makes comparative analysis more difficult. However, when comparing its biomass concentration after 10 days of cultivation with *N. oculate,* around 0.35 g/L was achieved, while the latter achieved a higher value, which is approximately 1.20 g/L. Furthermore, less biomass concentration was achieved without feeding CO_2_ to our cultivation process and a higher value was achieved when the ambient air was fed instead of CO_2_.

**FIGURE 2:**
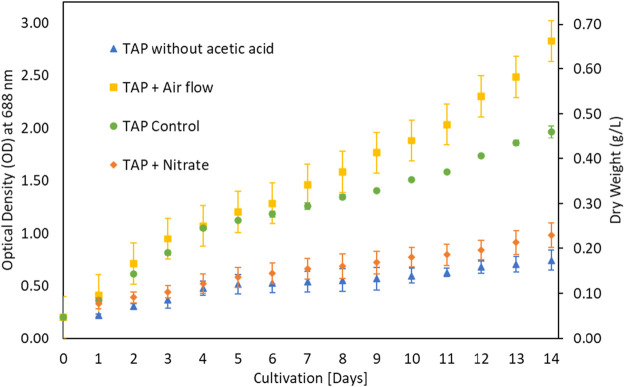
Supplementation variation in TAP medium.

The growth of microalgae for 10 days period (Ale et al., 2014) is shown in Figure 1 which implied that microalgae prefer low CO_2_ contents and do not prefer higher CO_2_ for its growth despite controlling pH. To investigate the possibility of microalgae preference for organic carbon, microalgae algae were cultivated in Triacetate Phosphate medium containing organic carbon (acetic acid and glucose) and without the organic carbon. Figure 2 showed that microalgae growth was affected by the presence of the organic carbon source in the form of acetic acid. A difference in growth is evident during growth without acetic acid. Purging the growth media with air in the presence of acetic acid further improved growth, which could be due to mixotrophic behavior and exposure of cells to lighter cells due to mixing (Gao et al., 2019; Patel et al., 2020). The use of organic waste and wastewater loaded with organic carbon will be a good cultivation medium for the growth of microalga, *G. pectorale,* because of its mixotrophic mode of growth.

**FIGURE 3:**
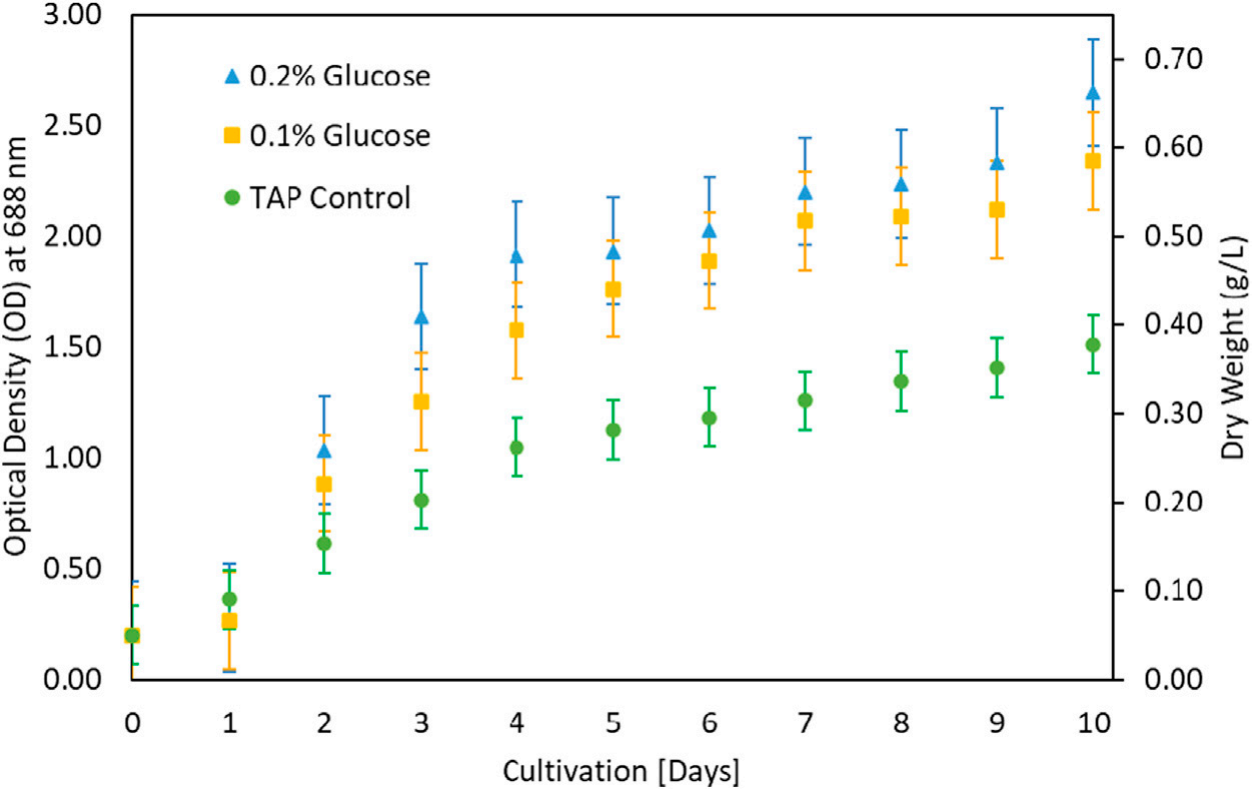
Growth comparison after glucose addition in TAP medium.

The growth of microalgae was further investigated at various glucose concentrations as a carbon source. The results in Figure 3 showed that growth improved with low glucose supplementation. Biomass increased from 0.4 g/L under control to 0.7 g/L under 0.2% glucose supplementation. The *G. pectorale* microalga was shown to be capable of consuming different types of organic carbon. TAP medium was preferred for glucose supplementation, unlike Modified Bold 3N medium. When the cultivation process was conducted in a modified Bold 3N medium with the addition of glucose, the culture growth failed. However, adding a higher glucose concentration (0.5%) to the TAP medium also resulted in growth failure. The biomass concentrations achieved in [g/L] are 1.01 ± 0.05, 1.10 ± 0.07, and 0.98 ± 0.08 for control, 0.1 and 0.2% glucose, respectively. For the same supplementation concentration and conditions, Karpagam et al. (2015) found the biomass concentration in [g/L] to be 0.77 ± 0.04, 0.81 ± 0.01, and 0.85 ± 0.03 for control, 0.1 and 0.2% glucose, respectively, for *Chlamydomonas reinhardtii*, *strain* CC1010 (Karpagam et al., 2015). In Figure 3, although 0.2% glucose supplementation improved *G. pectorale growth,* it does not make a noticeable difference compared to control growth using acetic acid as a source of organic carbon. Consumption of organic and inorganic form of carbon is supported by optimal light intensity, types and its duration (Mondal et al., 2017).

### 3.2 Ultimate analysis, CO_2_ fixation rate and higher heating value

The results of the ultimate analysis for the microalgae in Table 1 are consistent with a previous study (Yang et al., 2016; Farooq et al., 2022) in which most species of algae have a carbon content ranging from 40 to 50%, while the hydrogen content is approximately 7% of the total dry weight of the algal. The sulfur content in the microalgae is small, which ranges from 0.5% to 1.5%. The nitrogen content related to the microalgae protein content and amino acids ranges from 3.1% to 10.6%.

**TABLE 1:** Ultimate analysis and higher heating value of *G. pectorale* at different TAP media conditions.

	Ultimate analysis [wt%]					HHV [MJ/kg]	P [mg/L/Day]	R_CO_ [g/L/Day]
	C	H	N	S	O			
TAP + air	50.78	7.70	8.83	0.48	32.69	23.69	118.8	—
TAP + nitrate	48.43	7.72	8.78	0.57	35.07	22.67	84.7	—
TAP without acetic acid	43.17	6.55	7.0	0.96	43.28	18.42	68.3	—
TAP + 0.1% glucose	54.55	7.09	9.72	0	28.64	22.14	98.1	—
TAP + 0.2% glucose	45.99	6.53	8.30	0	39.18	18.08	110	—
TAP control	49.00	8.67	8.54	0.42	29.34	20.69	101	—
3NBBM control	47.19	7.49	7.75	0	37.57	19.01	65.4	—
3NBBM + air	47.18	6.49	8.16	0.05	38.17	18.62	72.8	—
3NBBM + 1% CO_2_	44.58	8.81	6.28	0	40.33	18.24	79.4	0.130
3NBBM + 2% CO_2_	49.34	7.04	8.64	0.69	34.98	20.42	74.2	0.134
3NBBM + 5% CO_2_	45.46	5.96	8.50	0.44	40.08	18.01	77	0.128

### 3.3 TG-DTG analysis of *G. pectorale* and pyrolytic kinetics

**FIGURE 4:**
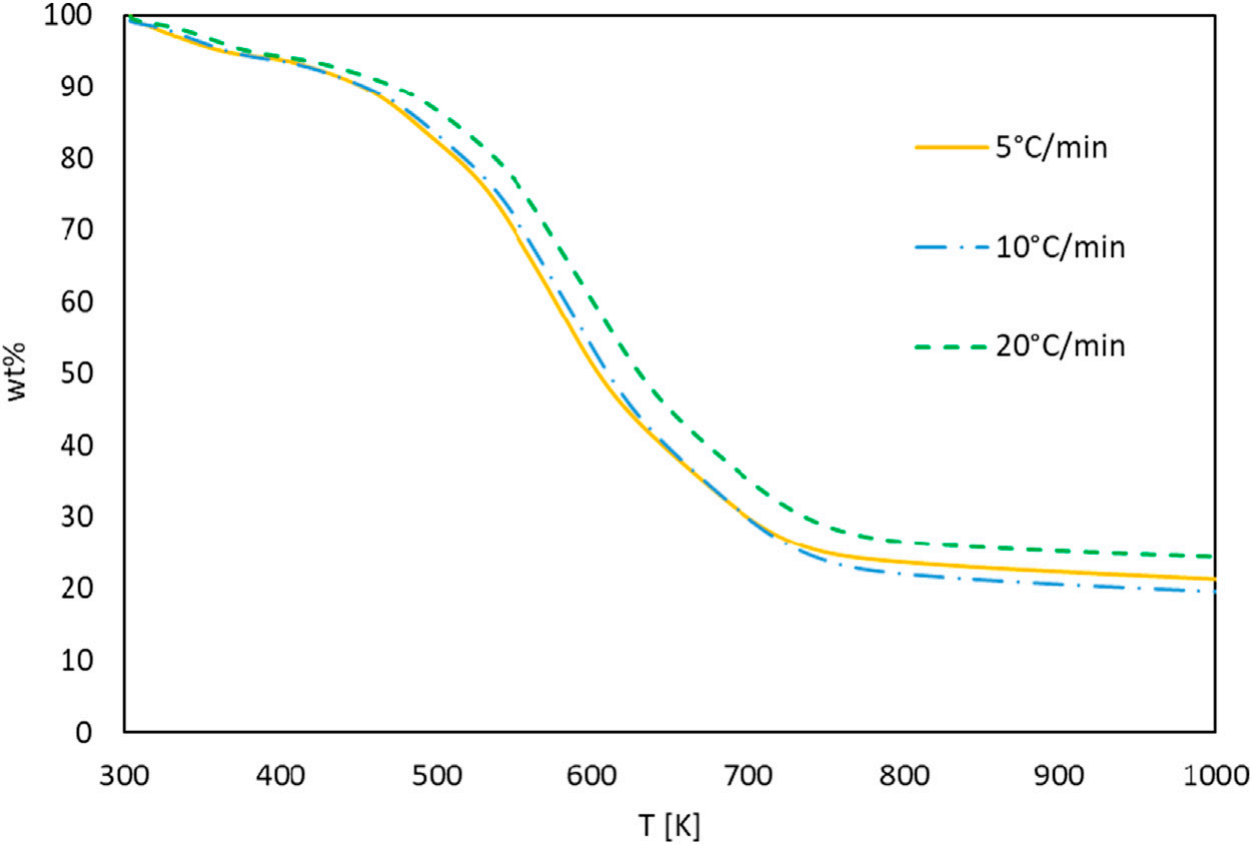
TG curves of *G. pectorale* at different heating rates.

**FIGURE 5:**
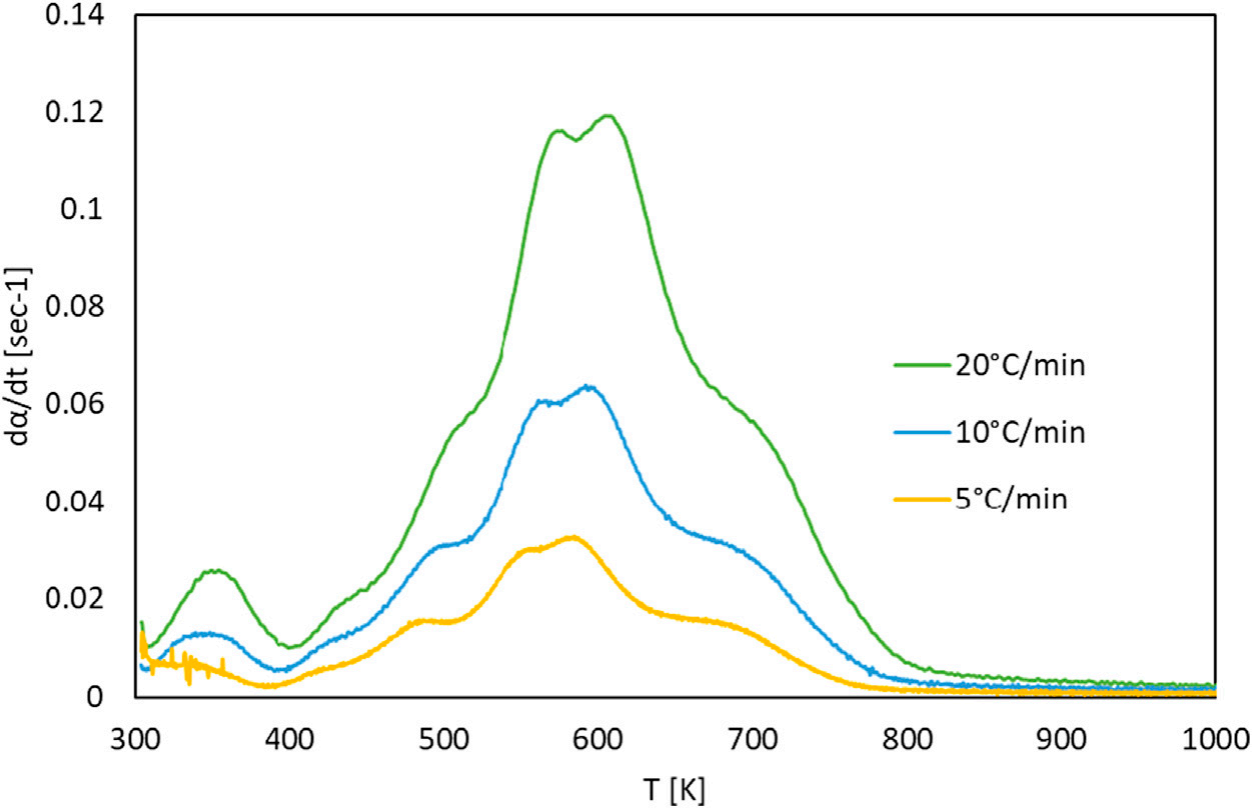
DTG curves of *G. pectorale* at different heating rates.

The TG and DTG curves of the *G. pectorale* at 5, 10, and 20°C/min are shown in Figures 4, 5. It is noticeable that an increase in the heating rate results in an increase in both the degradation rate (dα/dt) and the releases of volatile matter and in fewer pyrolysis residues (Subagyono et al., 2021). This is due to the limitation of mass and heat transfer that is normally attributed to high temperatures (Ceylan et al., 2014). Figure 4 shows a significant weight loss in the temperature range of 540–740 K. In addition, even the DTG peaks shifted to higher temperatures. The DTG curves at 5, 10, and 20°C/min indicated the presence of multiple peaks and pyrolysis zones, with the maximum peaks’ temperatures shown in Table 2 that correspond to the devolatilization process or the main pyrolysis. These peaks are generally attributed to the decomposition of proteins and carbohydrates (Vuppaladadiyam et al., 2019). Three stages can be clearly shown on the TG-DTG curves. The first stage is at ≤ 400 K, where evaporation of moisture and low-boiling point organic compounds occurs. Furthermore, chlorophyll decomposition can occur during this stage because it is an unstable compound that generally degrades at 80°C–145°C (Chen et al., 2012). The second stage lies between 400 and 740 K, is the active pyrolysis zone where the thermal decomposition of carbohydrates, proteins and lipids occurs at 410–540 K, 470–550 K, and 560–630 K, respectively. The third stage, known as the passive pyrolysis zone, is indicated by flat curves that are higher than 760 K, where the decomposition of the compounds occurs due to gasification and non-volatile carbon compounds that evaporate to form gaseous CO_2_ and CO at high temperature (Agrawal and Chakraborty, 2013).

**TABLE 2:** Characteristics of DTG curves at different heating rates of *G. pectorale* pyrolysis.

β [°C min^−1^]	T_p_ [K]	dα/dt [sec^−1^] |_Tp_
5	592.72	0.031
10	593.62	0.063
20	606.99	0.119

### 3.4 Model-free kinetics

**FIGURE 6:**
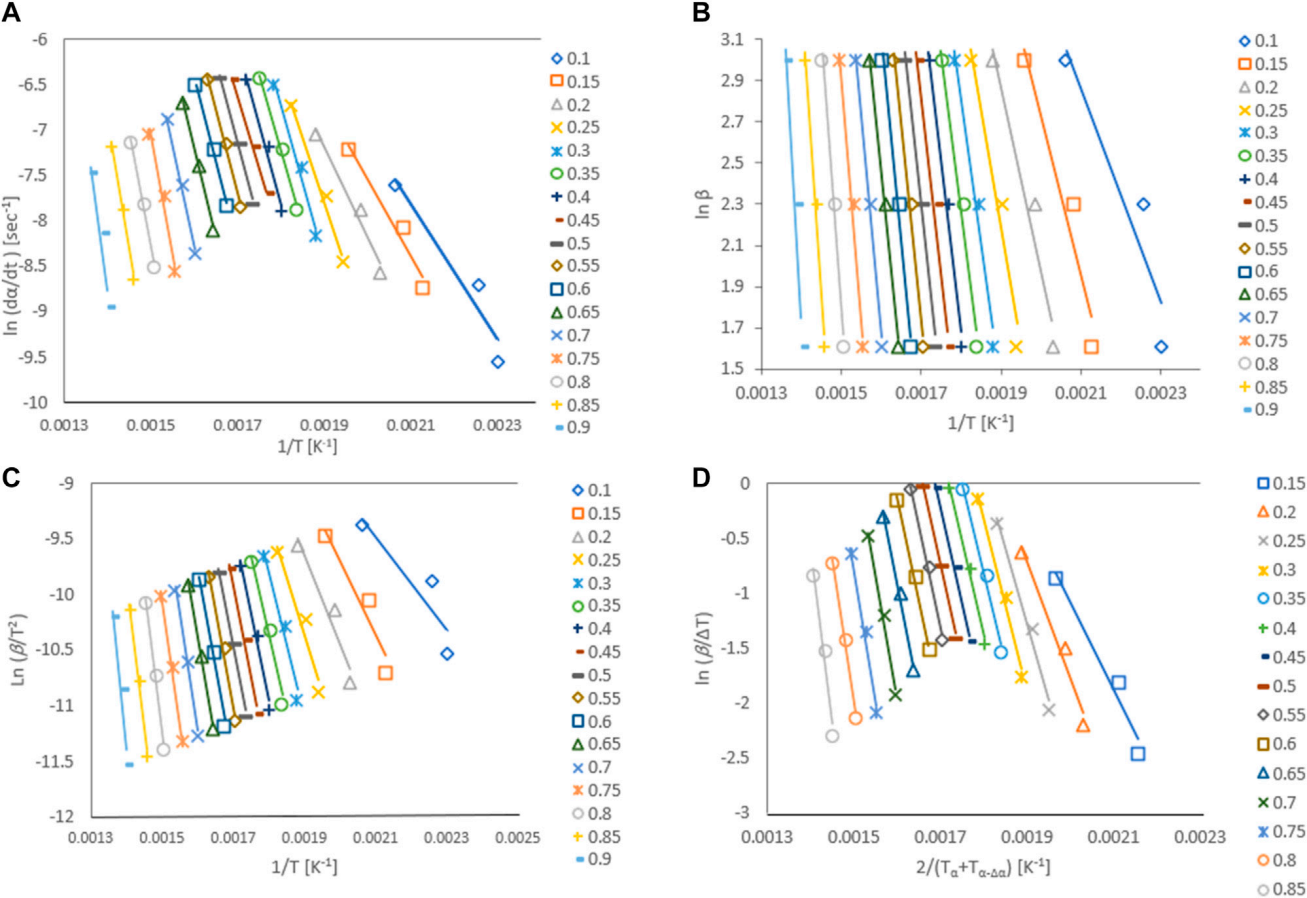
Arrhenius plots for *G. pectorale* at different kinetic models **(A)** Friedman, **(B)** Flynn-Wall-Ozawa (FWO), **(C)** Kissinger-Akahira-Sunose (KAS), and **(D)** Popescu.

**FIGURE 7:**
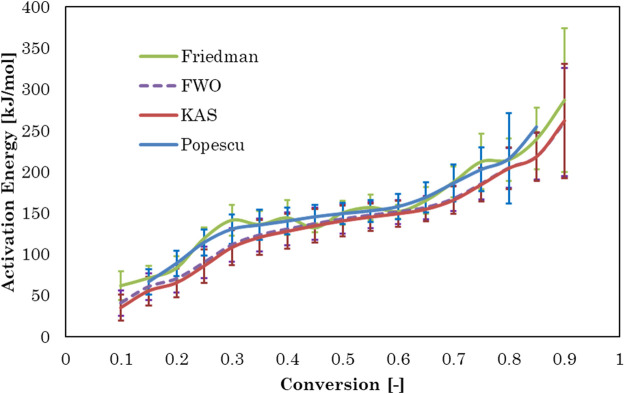
Activation energy versus conversion for four model-free methods.

Activation energy, Ea, is the minimum energy required in order to form a product. The value of activation energy can be found from Arrhenius plot. Arrhenius plot analyze the effect of reaction temperature on rate of reaction. The Arrhenius plots for Gonium pectorale are plotted for different kinetic models as Figure 6. The estimated values of the activation energy, which depend on the composition of the biomass, for the pyrolysis of the microalgae biomass are given in Figure 7. The obtained *Ea* values were calculated for the conversion range of 0.1–0.8 with a step interval of 0.05. The relative contents of lipids, carbohydrates, and protein and their classes vary between microalgae strains. A higher standard deviation at the end might be due to the presence of ash contents containing minerals. Furthermore, calculated activation energy values using FWO (40.6–260 kJ/mol), Friedman (61.7–287 kJ/mol), Popescu (66.4–255 kJ/mol) and KAS (35.0–262 kJ/mol), respectively. The estimated values of activation energies from different models at multiple heating rates of 5, 10 and 20°C/min are in close agreement with each other and agree with the reported literature. (Farooq et al., 2021). estimated activation energy for the pyrolytic conversion of *Parachlorella kessleri* HY-6 using KAS and Friedman methods as 241.91 (±53.05) kJ/mol and 253.54 (±58.81) kJ/mol, respectively. In another study, the authors calculated activation energy for Spirulina pyrolysis using KAS method in the range of 160–335 kJ/mol (Hong et al., 2020).

Activation energy values using the KAS, FWO, Popescu, and Friedman methods for conversion rates from 0.1 to 0.3 increased due to protein decomposition in this conversion temperature range (233°C–340°C). A slight decrease in the activation energy value was observed at conversion rates of 0.4–0.6, which showed cellulose decomposition in the temperature range (326°C–393°C). Pyrolytic degradation of lipid compounds required higher activation energy values conversion rates of 0.6–0.9, in a higher temperature range of 377°C–484°C (Vuppaladadiyam et al., 2019; Subagyono et al., 2021). Similar observation was reported in another study, where model compound of protein decomposed earlier at lower temperature than carbohydrates and then lipid (Hong et al., 2020).

### 3.5 Thermodynamics parameters estimation

Endothermic or exothermic nature of the reaction is indicated by ΔH. The value of ΔH also indicates the energy difference between the activated complex and the reactants. Small ΔH represents the formation of activated complex and a low potential energy barrier (Naqvi et al., 2018). The average ∆H was 135.82 (±57.58) kJ/mol and the difference between Ea and ∆H is around 5 kJ mol^−1^. ∆G refers to the increase in the energy of the system towards an equilibrium by forming activated complex. ΔG values range from (61.2–250 kJ/mol), (168–384 kJ/mol), (176–214 kJ/mol), and (121–162 kJ/mol) for the Popescu, KAS, FWO, and Friedman methods, respectively. These ΔG values indicate the increase in total energy available in *G. pectorale* pyrolysis and the formation of an activated complex. Furthermore, these values are higher compared to the values of waste red peppers (139.0 kJ/mol) (Maia and de Morais, 2016) and rice straw (165.1 kJ/mol) (Xu and Chen, 2013). ∆S indicates the degree of proximity of the system to thermodynamic equilibrium. Lower values of ∆S indicate that material passed a process, moving to a thermodynamic equilibrium, while higher ∆S values states that the material is away from thermodynamic equilibrium. The negative value of ΔS and the positive value of ΔG indicated in Table 3 imply that the thermal decomposition of *G. pectorale* is a non-spontaneous process. When R^2^ is close to 1, this indicates that we have an excellent fit model to the experimental data from TG. However, a higher R^2^ of fit is not always a suitable criterion to decide which methods are best because it does not determine whether the activation energies are correct (Subagyono et al., 2021).

**TABLE 3:** *G. pectorale* thermodynamic parameters by Flynn-Wall-Ozawa (FWO) method.

Conversion	E_a_ [kJ/mol]	R^2^	A [min^−1^]	∆H [kJ/mol]	∆G [kJ/mol]	∆S [kJ/mol/K]
0.1	40.6	0.88	5.14E + 01	35.4	175.9	−0.227
0.15	60.3	0.93	5.22E + 03	55.2	171.8	−0.188
0.2	70.0	0.95	3.05E + 04	64.8	172.4	−0.173
0.25	89.6	0.96	2.06E + 06	84.5	170.3	−0.138
0.3	111.2	0.97	1.97E + 08	106.0	168.3	−0.101
0.35	123.1	0.97	1.85E + 09	118.0	168.8	−0.082
0.4	130.3	0.98	5.51E + 09	125.2	170.3	−0.073
0.45	136.9	0.98	1.37E + 10	131.7	172.2	−0.065
0.5	142.7	0.98	2.80E + 10	137.5	174.3	−0.059
0.55	147.3	0.99	4.36E + 10	142.1	176.6	−0.056
0.6	151.3	0.99	5.71E + 10	146.1	179.2	−0.053
0.65	156.6	0.99	9.12E + 10	151.5	182.2	−0.050
0.7	167.4	0.99	3.71E + 11	162.2	185.7	−0.038
0.75	185.6	0.99	5.12E + 12	180.4	190.3	−0.016
0.8	204.8	0.99	6.66E + 13	199.7	196.4	0.005
0.85	218.7	0.98	2.51E + 14	213.5	203.4	0.016
0.9	260.3	0.94	9.88E + 16	255.2	214.3	0.066

### 3.6 Model-fitting kinetics

**FIGURE 8:**
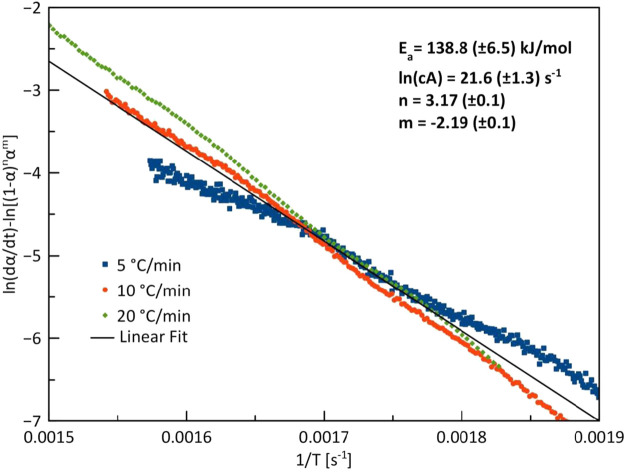
Combined kinetics plot of *G. pectorale* at heating rates of 5, 10, and 20°C/min.

Figure 8 shows the kinetics plots of the conversion of *G. pectorale* under slow pyrolytic conditions with three different heating rates. At 10 and 20°C/min, the heating rates yielded near-straight lines that fit the experimental curve. However, at 5°C/min, the line produced differs from the experimental curve.

**FIGURE 9:**
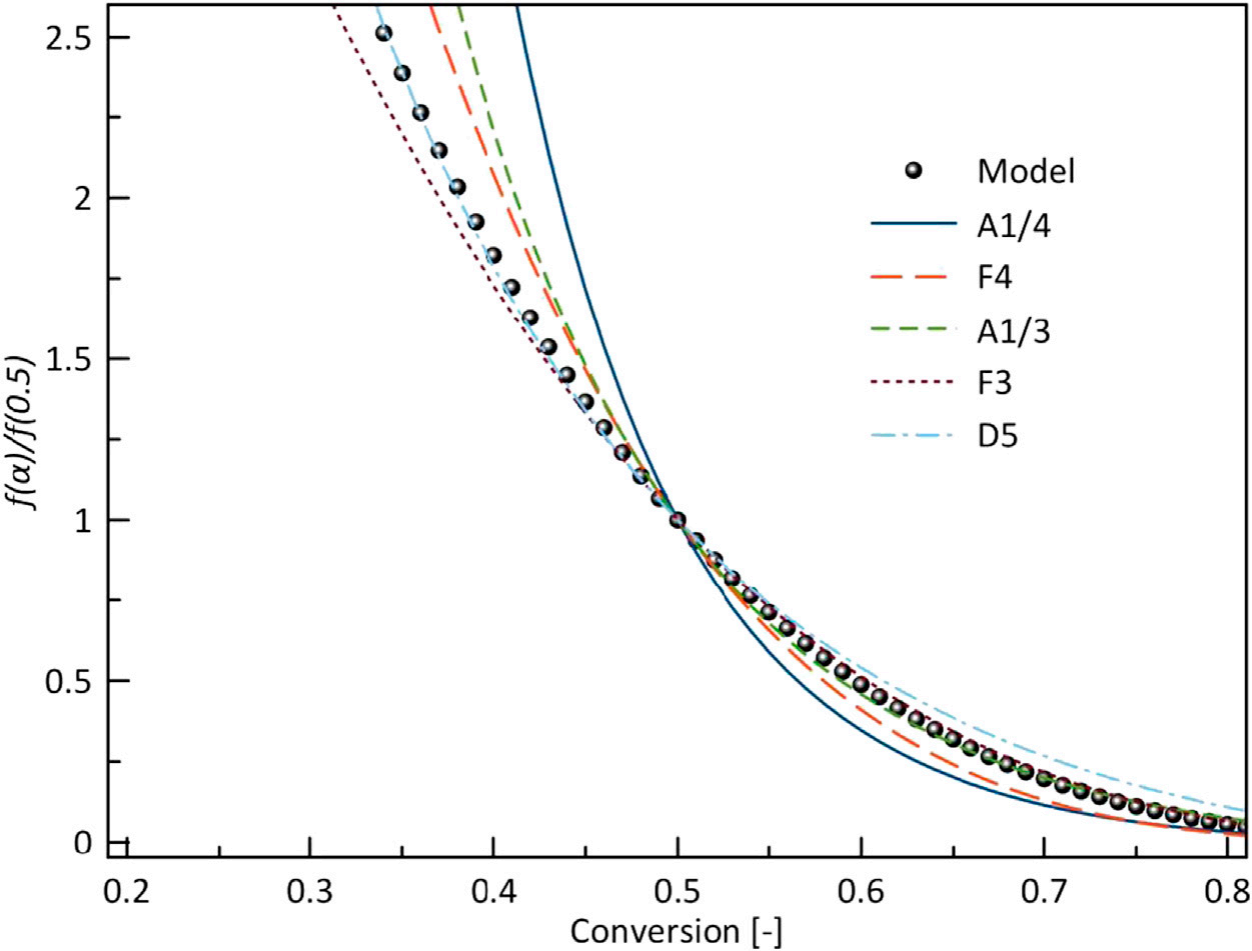
Normalized model function *f(α)/f(0.5)* with different ideal models.

Figure 9 shows model-fitting kinetics with master plot agreement with five different models. These models are the most likely reaction mechanism for a single-step reaction. A third-order (F3) corresponded to the combined kinetic parameters obtained in this study. The obtained kinetics order at different heating rates is shown in Table 4, the order is slightly lower than reported by (Alshareef and Ali, 2020) in the pyrolysis of halophyte. By the high reaction order indicates random nucleation within the particles during pyrolysis (Xu et al., 2017).

**TABLE 4:** Summarizes the combined kinetics plots of *G. pectorale* at three different heating rates.

Heating rate [^o^C/min]	E_a_ [kJ/mol]	ln(cA) [1/s]	n [−]	m [−]
5	71.5 ± 2.5	9.7 ± 0.5	3.38 ± 0.04	0.4 ± 0.04
10	99.9 ± 3.3	15.6 ± 0.7	3.61 ± 0.06	−0.43 ± 0.06
20	82.6 ± 9.6	12.1 ± 1.9	3.07 ± 0.18	−0.18 ± 0.18

### 3.7 Artificial neural network prediction

MLP based ANN regression models were trained and five better performing models were retained. The most suitable model was selected on the basis of the highest correlation coefficient and lowest sum of squared errors during training, testing and validation of the data sets. The structure of the best performing network at different heating rates was MLP 4-10-1. MLP based ANN model containing 10 hidden layers with exponential function in the hidden layers and sine function in the output layer returned excellent correlation coefficient. MLP 4-10-1 was therefore used to understand the microalgae biomass conversion process through pyrolysis. The BFGS algorithm for the said model reached optimal outcomes after 62 cycles.

**FIGURE 10:**
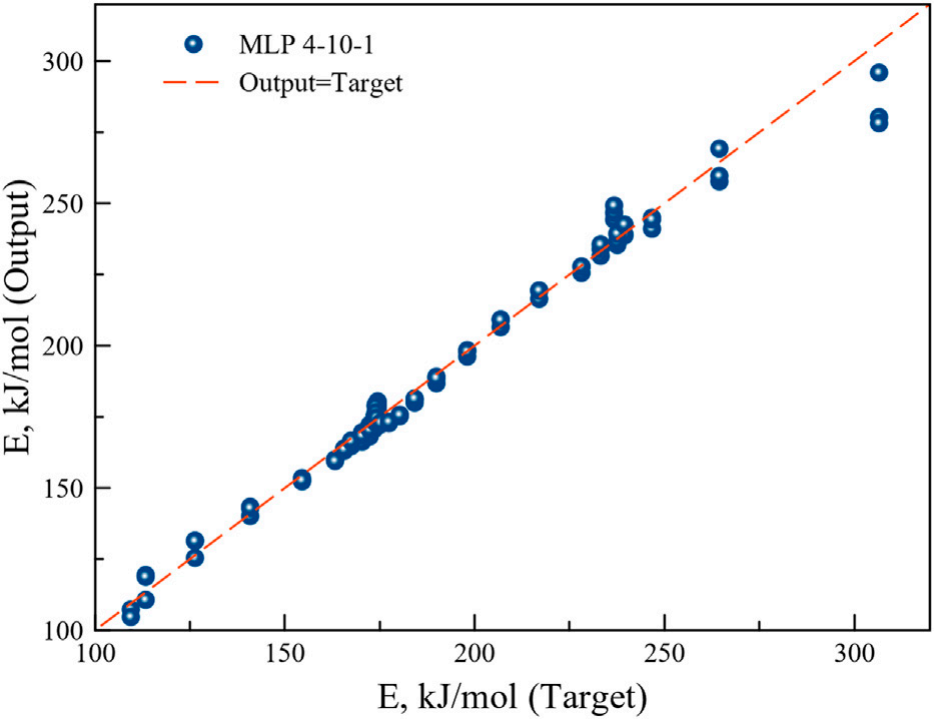
Regression plot of the ANN model output with the experimental target.

The regression graph in Figure 10 shows the correlation between the target and the model output values. The high correlation coefficient implies good agreement of the model output with the experimental target values.

**FIGURE 11:**
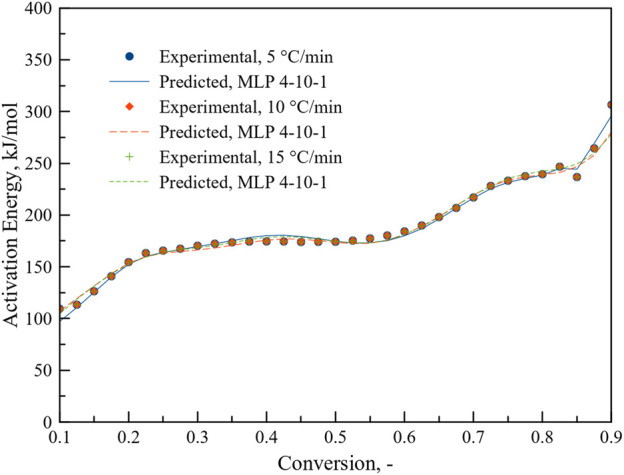
Prediction of activation energy as a function of conversion at different heating rates.

To see the performance, MLP 4-10-1 was used to predict the activation energy at different heating rates. As can be seen from Figure 11, MLP based ANN regression model can predict activation energy accurately for different heating rates as a function of conversion. R^2^ value reached to 0.999. MLP based ANN performed better than ANN results reported in a recent study on the thermal degradation of green river shale (You and Lee, 2022) where heat flow, heating rate and difference in conversion were chosen as inputs. There model performance was relatively poor at lower heating rate.

## 4 Conclusion

*G. pectorale* was grown to analyze its growth, CO_2_ fixation capacity, thermogravimetric analysis (TG), derivative thermogravimetric analysis (DTG), and elemental analysis to estimate its energy content. Growth was carried out under different culture conditions in two different media, reporting the highest biomass productivity, and the CO_2_ fixation rate was obtained from TAP culture and 3NBBM with a concentration of 2% CO_2_, respectively. The highest biomass concentration achieved after 14 days of cultivation was 1.08 g/L. As a result, TAP is considered a better medium compared to 3NBBM for the cultivation of *G. pectorale.* This shows that *G. pectorale* prefers organic carbon over inorganic carbon (CO_2_). TG-DTG curves at 5, 10, and 20°C/min indicated the presence of multiple peaks and active pyrolysis zones due to the multicomponent biomass of microalgae (carbohydrates, protein, and lipids). In addition, the kinetics of *G. pectorale* was studied using model-free and model-fitting methods. The predicted activation energy values of the Friedman, FWO, KAS and Popescu models indicated excellent agreement with the experimental values (R^2^ > 0.96). The higher values of 200 kJ/mol of *Ea* obtained suggest that algal lipids are more difficult to decompose in the N_2_ atmosphere. Moreover, it was found that the most probable reaction mechanism for the pyrolysis of *G. pectorale* was the third-order function. Also, the F3 model-fitting method gave a good prediction. The study showed the effectiveness of MLP based ANN regression model for the prediction of activation energy at different heating rates. Further investigations must be conducted on the heterotrophic and mixotrophic cultivation using different organic molecules for lipids production. Detailed characterization of the strain with respect to its biomolecules is also recommended. As this microalga showed a preference for organic carbon, it is recommended to use this strain for treatment of high organic load wastewater.

## Data Availability

The original contributions presented in the study are included in the article/Supplementary Material, further inquiries can be directed to the corresponding author.
