# Supplementary material for: Assessment of CO2 biofixation and bioenergy potential of microalga Gonium pectorale through its biomass pyrolysis, and elucidation of pyrolysis reaction via kinetics modeling and artificial neural network
Source: Front Bioeng Biotechnol. 2022 Aug 18;10:925391. doi: 10.3389/fbioe.2022.925391 (PMC9434281; doi:10.3389/fbioe.2022.925391)
Supplement: Supplementary file 1 [file DataSheet1.docx]

# Appendix A. Supplementary material

Figure 11. Observed pH trend during *Gonium pectorale* cultivation period for 15 days in TAP medium.

| **Stock solution** | **Amount (mL/2L)** | **Component** | **Amount (g/L)** | **Concentration (mM)** |
| --- | --- | --- | --- | --- |
| **Beijerinck's Solution** | **100** | **NH_4_Cl** | **8** | **7.5** |
|  |  | **CaCl_2_ ·2H2O** | **1** | **0.35** |
|  |  | **MgSO_4_ ·7H_2_O** | **2** | **0.4** |
| **Phosphate Buffer Stock Solution** | **17** | **Na_2_HPO_4_** | **11.62** | **0.69** |
|  |  | **KH_2_PO_4_** | **7.26** | **0.45** |
| **Hunter's Trace Stock Solution** | **2** | **Na_2_ EDTA·2H_2_O** | **50** | **0.134** |
|  |  | **ZnSO_4_ ·7H_2_O** | **22** | **0.077** |
|  |  | **H_3_BO_3_** | **11.4** | **0.184** |
|  |  | **MnCl_2_ ·4H2O** | **5.1** | **0.026** |
|  |  | **FeSO_4_·7H2O** | **5** | **0.018** |
|  |  | **CoCl_2_·6H_2_O** | **1.6** | **0.007** |
|  |  | **CuSO_4_·5H_2_O** | **1.16** | **0.005** |
|  |  | **(NH_4_)_6_Mo_7_0_24_ ·4H_2_O** | **1.1** | **0.0008** |
| **Tris Acetate Stock Solution** | **20** | **Trisma Base** | **242** | **20** |
|  |  | **Glacial Acetic Acid** | **100** | **17** |

Table 5. Tris-Acetate-Phosphate medium recipe for 2 Liter volume.

Table 6. Modified Bold 3N medium recipe.

| **Component** | **Amount (mL/L)** | **Stock solution concentration (g/400ml)** | **Concentration (mM)** |
| --- | --- | --- | --- |
| **NaNO_3_** | **30** | **10** | **8.82** |
| **CaCl_2_·2H_2_O** | **10** | **1** | **0.17** |
| **MgSO_4_ · 7H_2_O** | **10** | **3** | **0.3** |
| **K_2_HPO_4_** | **10** | **3** | **0.43** |
| **KH_2_PO_4_** | **10** | **7** | **1.29** |
| **NaCl** | **10** | **1** | **0.43** |
| **P-IV Metal Solution** | **6** |  | |
| **Soilwater: GR+ Medium** | **40** |  |  |
| **Vitamin B12** | **1** |  |  |
| **Biotin Vitamin Solution** | **1** |  |  |
| **Thiamine Vitamin Solution** | **1** |  |  |
